# Mental health after first childbirth in women requesting a caesarean section; a retrospective register-based study

**DOI:** 10.1186/s12884-017-1514-2

**Published:** 2017-09-29

**Authors:** Louise Möller, Ann Josefsson, Marie Bladh, Caroline Lilliecreutz, Ellika Andolf, G. Sydsjö

**Affiliations:** 1 Department of Obstetrics and Gynaecology, and Department of Clinical and Experimental Medicine, Linköping University, SE-581 85 Linköping, Sweden; 2 Division of Obstetrics and Gynaecology, Department of Clinical Sciences, Karolinska Institutet, Danderyd Hospital, Stockholm, Sweden

**Keywords:** Psychiatric disorders, Caesarean section, Maternal request

## Abstract

**Background:**

Psychiatric illness before delivery increases the risk of giving birth by caesarean section on maternal request (CSMR) but little is known about these women’s mental health after childbirth. In this study we aimed to compare the prevalence of psychiatric disorders five years before and after delivery in primiparae giving birth by CS on maternal request to all other primiparae giving birth, indifferent on their mode of delivery.

**Methods:**

The study population comprised all women born in Sweden 1973–1983 giving birth for the first time in 2002–2004. Psychiatric diagnoses, in- and outpatient care were retrieved from the National Patient Register in Sweden. The risk of psychiatric care after childbirth was estimated using CSMR, previous mental health and sociodemographic variables as covariates.

**Results:**

Psychiatric disorders after childbirth were more common in women giving birth by CSMR compared to the other women (11.2% vs 5.5%, *p* < 0.001). CSMR increased the risk of psychiatric disorders after childbirth (aOR 1.5, 95% CI 1.2–1.9). The prevalence of psychiatric disorders had increased after compared to before childbirth (mean difference 0.02 ± 0.25, 95% CI 0.018–0.022, *p* < 0.001). Women giving birth by CSMR tended to be diagnosed in the inpatient care more often (54.9% vs. 45.8%, *p* = 0.056) and were more likely to have been diagnosed before childbirth as well (39.8% vs. 24.2%, *p* < 0.001).

**Conclusions:**

Women giving birth by CSMR more often suffer from psychiatric disorders both before and after delivery. This indicates that these women are a vulnerable group requiring special attention from obstetric- and general health-care providers. This vulnerability should be taken into account when deciding on mode of delivery.

## Background

The rate of caesarean sections (CS) is increasing with an average rate of 15% worldwide and 21% in developed countries [1]. One reason for this trend is an increase in the frequency of CS on maternal request (CSMR). In Stockholm, Sweden, CSMR increased from 10.5% of all elective CS in 1992 to 38.5% in 2005, i.e. 4% of all deliveries [2]. Fear of childbirth (FOC) is the most common reason given for CSMR [3–7]. FOC affects approximately 7–15% of all pregnant women [8–11]. The fear may be due to a previous negative birth experience, obstetric complications such as an emergency CS/vacuum extraction [5, 8, 9], lack of trust in the obstetrical staff, loss of control, pain, as well as fear of one’s own death and/or the infant’s death [12]. Maternal characteristics associated with the preference for a CS are higher maternal age, smoking, lower educational attainment, unemployment [3, 5], living alone [5], personality, and being an immigrant [13].

These women giving birth by CSMR (hereafter CSMR women) suffer more often from psychiatric illness than women giving birth by other modes of delivery. Mood disorders, anxiety [10, 14, 15], and the use of psychotropic medications are more common in women fearing childbirth [11, 14]. We have earlier reported that the presence of psychiatric disorders five years before childbirth increases the risk of giving birth by CSMR (aOR 2.5, 95% CI 2.0–3.2) [16]. The most common disorders were mood-, anxiety disorders and mental and behavioural disorders due to psychoactive substance use [16]. To our knowledge, no one has studied the long-term mental health after childbirth of CSMR women. Because having had psychiatric care before pregnancy and FOC have both been shown to increase the risk of requiring psychiatric care after delivery [14], we hypothesize that CSMR women are more likely to suffer from an increased psychiatric disease burden after delivery than any other group.

Therefore, the aim of this study was to compare the prevalence of psychiatric care five years before and after delivery in primiparae giving birth by CSMR with prevalence in all other primiparae giving birth during the study period. This knowledge is important in order to provide optimal care for these women. By using the identical study population as in our previous study [16], we have been able to compare the prevalence of psychiatric disorders before and after delivery. The study population comprises a nationwide cohort of all women born in Sweden 1973–1983, giving birth for the first time in 2002–2004.

## Methods

The study population is identical to the study population in our previous study and the methods for collecting the data have been described before [16].

The material is based on data from several Swedish National registries. By using the women’s unique personal identification number, data from several registries were linked. Assigning every woman a new de-identified number anonymised the dataset.

The Swedish Medical Birth Register (MBR) contains information on maternal characteristics, pregnancies, deliveries and birth characteristics. The MBR was begun in 1973 and has high validity, covering approximately 99% of all deliveries [17]. From this register mode of delivery and maternal characteristics at admission to antenatal care (age, BMI, tobacco use and somatic disorders) were gathered. The National Patient registry (NPR) started to record inpatient care in 1987 and from 2001 and onwards it also contains outpatient care except for primary health care. The coverage of the inpatient care is generally high with 90–94% having a main diagnosis recorded during the study period. The data from the outpatient care are less reliable with 34–79% having a main diagnosis registered during the study period [18]. The outcome of interest in this part of the study was psychiatric illness five years after delivery. Psychiatric illness was defined as in- and/or outpatient care registered together with a psychiatric diagnosis according to the International Classification of Diseases (ICD). Psychiatric diagnoses were identified as codes F00-F99 and these codes were divided into 11 categories according to ICD-10 (Table 1) [19, 20]. The time period was defined as date of first delivery plus five years. Only dates of admission or dates of visit during this timeframe were included. The prevalence of psychiatric illness five years before delivery has been published in our previous study and was retrieved using the same methods [16].

**Table 1:** Categories of psychiatric disorders according to ICD-10

F00-F09	Organic, including symptomatic, mental disorders
F10-F19	Mental and behavioural disorders due to psychoactive substance use
F20-F29	Schizophrenia, schizotypal and delusional disorders
F30-F39	Mood (affective) disorders
F40-F48	Neurotic, stress-related and somatoform disorders
F50-F59	Behavioural syndromes associated with physiological disturbances and physical factors
F60-F69	Disorders of adult personality and behaviour
F70-F79	Mental retardation
F80-F89	Disorders of psychological development
F90-F98	Behavioural and emotional disorders with onset usually occurring in childhood and adolescence
F99-F99	Unspecified mental disorder

Background data on sociodemographic variables were gathered from the Total Population register (TPR) [21], the Education register [22], and the Multi-generation register [23]. TPR contains data on births, deaths, citizenship, marital status, migration and country of birth for Swedish residents born abroad [21]. The Education register records data on highest educational attainment and the multi-generation register records kinship [22]. By using the multi-generation register we could identify the parents of our study sample [23].

The study population comprised all women born in Sweden 1973–1983 who were alive and still living in Sweden at 13 years of age (*n* = 500,245). The original dataset was sorted based on data from the Causes of Death registry, which has reported on causes of deaths since 1961 and has a high validity [24]. For the purpose of another study using the same dataset, women with missing values on birthweight, gestational length as well as implausible birthweights compared to length of gestation at their own birth were excluded (*n* = 5553). From the final cohort we selected women giving birth for the first time in 2002 to 2004 (*n* = 64,834). Only primiparae were included because we were interested in primary FOC.

The exposure of interest was CSMR. CSMR was defined as the delivery diagnosis O82.8 in ICD-10 [10, 20]. In the Swedish version of ICD-10 it is labelled “Caesarean section on psychosocial indication” and it is the code that should be used for CSMR [19] (labelled as “encounter for caesarean without indication” in the English version of ICD-10) [20]. However, we do not know whether the women actually requested a CS.

CSMR women (*n* = 1009, 1.6%) were compared to women giving birth by other modes of delivery (*n* = 63,826) (hereafter referred to as “the reference group”) including vaginal delivery (*n* = 55,012, 84.9%), emergency CS (*n* = 5826, 9%) and elective CS for all other reasons except maternal request (*n* = 2859, 4.4%). Vaginal delivery was defined as ICD-10 codes; spontaneous vaginal delivery (O80), single instrumental vaginal delivery by forceps or vacuum extraction (O81), other assisted single delivery (O83) or multiple vaginal delivery (O84.1 and O84.9). Included were also women missing a delivery diagnosis but having the diagnosis “care after vaginal delivery” (Z.39.0A/B) or a notation in MBR that the delivery ended “vaginally”, “by forceps” or “by vacuum extraction”. Emergency CS was defined as single (O82.1) and multiple delivery (O84.8) by emergency CS or a notation in MBR that the delivery was by “emergency CS”, “not an elective CS” or “delivery ended by CS”. Elective CS single delivery (O82, O82.0, O82.9) and multiple delivery by elective CS (O84.2 and O84.3) as well as women missing delivery diagnosis but having the diagnosis “care after CS” (Z.39.0C/D) or a notation in MBR saying “delivery by elective CS before contractions started”, “delivery by elective CS” or “delivery started by CS”. The following background variables were registered in MBR at admission to antenatal care; age, body mass index (BMI), smoking status, the use of snuff, and somatic diseases (recurring urinary tract infection, inflammatory bowel disease, epilepsy, asthma/lung disease, diabetes mellitus, kidney disease, hypertension and systemic lupus erythematosus). Age was categorized into ≤25 or >25 years old. BMI was divided into five categories (<18.5, 18.5–24.9, 25–24.9, 25–29.9, 30–34.9 and ≥35 kg/m^2^). Smoking and the use of snuff were collapsed into one variable called “tobacco use” which thus includes the use of snuff and/or smoking. From the other registers, marital status, educational attainment, parents’ country of birth and working status were gathered. Marital status was categorized into “married”, “unmarried” and “divorced/widowed”. Highest educational attainment was assessed in 2004 and divided into three categories; “Elementary”, “High School” and “University”. Parents’ country of birth were categorised as born inside or outside of Scandinavia. Working status was defined as an income >39,300 SEK per year in 2004 according to Statistics Sweden’s definition of paid work.

### Missing data

Mode of delivery was missing for 128 (0.2%) women. Educational attainment was missing for 193 (0.3%) women. Working status was missing for 92 (0.1%) and BMI was missing for 8765 (13.5%) of women. Missing data were included in the analysis and applied their own category in each variable.

### Background data

Comparisons of sociodemographic variables and psychiatric illness five years before delivery between CSMR women and the reference group have been published earlier [16]. CSMR women were older (68.4 vs. 64.7% >25 years, *p* < 0.001), used tobacco more often (18.3 vs. 13.0%, *p* < 0.001), had a lower educational attainment (16.0 vs. 10.1% elementary school, *p* < 0.001), were more often married (36.2 vs 32.8%, p < 0.001), unemployed (13.1 vs 8.6%, p < 0.001), had a higher BMI (*p* < 0.001) and more often parents born outside of Scandinavia (9.1 vs 6.0%, *p* < 0.001) compared to the reference group. They had also more often recurrent urinary tract infection (22.5 vs 16.8%, p < 0.001), asthma/lung disease (13.1 vs 9.2%, p < 0.001) and inflammatory bowel disease (1.4 vs 0.7%, *p* = 0.01). No significant differences were found for kidney disease (0.8 vs 0.5%), diabetes mellitus (0.9 vs 0.5%), epilepsy (1.1 vs 0.6%), SLE (0.1 vs 0.1%) or hypertension (0.3 vs 0.3%) compared to the reference group.

### Statistics

CSMR women were compared to the reference group. The outcome of interest was psychiatric illness five years after first childbirth. Each category of psychiatric illness was treated as a binary variable. A binary variable for one or more registered psychiatric diagnosis in the in- and/or outpatient care (“any psychiatric disorder”) was constructed. A similar variable was created for outpatient care only and another variable including those diagnosed in the inpatient care only and/or both the in- and outpatient care. The prevalence of each category of psychiatric disorder, the distribution of in- and outpatient care as well as the prevalence of any psychiatric disorder in CSMR women was compared to the reference group five years after delivery using Pearson’s Chi square test. Whenever ≥20% of the expected values were below five, Fishers exact test was used instead. The prevalence of psychiatric disorders five years before delivery was compared to five years after delivery in the whole study population as well as within the two groups respectively by paired T-test. A paired T-test was considered appropriate since the sampling distribution of binary variables becomes approximately normally distributed in large samples according to the Central Limit Theorem.

To compare the rate of new cases (i.e. women diagnosed with a psychiatric disorder after first childbirth who had not been diagnosed during the five years before first childbirth), a binary variable was constructed for any psychiatric disorder after delivery that excluded cases diagnosed before delivery. Comparisons between the groups were made by Pearson’s Chi Square test in the whole study population as well as only for women diagnosed with a psychiatric disorder after childbirth to compare the proportion of new cases among the cases.

The risk of psychiatric illness after delivery was estimated by multiple logistic regression analysis. Psychiatric disorders five years after delivery was considered the outcome and CSMR the exposure. To adjust for potential confounding, previous psychiatric care and background variables (age, BMI, educational attainment, tobacco use, marital status, parents’ country of birth, working status and somatic diseases) were added to the model as covariates. Any psychiatric disorder as well as each of the eleven categories of psychiatric disorders were modelled separately in the logistic regression. CSMR and previous psychiatric care were tested for interaction effect in the logistic regression model. No significant interaction effect was found. A sensitivity analysis excluding all women diagnosed with a psychiatric disorder before first delivery was performed and no significant differences were found. All analyses were carried out in SPSS 22.0 (IBM SPSS Inc., Armonk, NY, USA). A *p*-value <0.05 was considered significant.

## Results

Of all the women in the study, 2316 (3.6%) women had been diagnosed with one or more psychiatric disorder during the five years before first childbirth [16] and 3607 (5.6%) women had been diagnosed during the five years after first childbirth. Eight hundred ninety-two women had been diagnosed both before and after first childbirth. During the five years after the first childbirth, 1946 women were diagnosed only in the outpatient care register and 1661 women were diagnosed in both the in- and outpatient care register or in the inpatient care register only. Women in the reference group tended to more often be diagnosed only in the outpatient care register (54.2% vs. 45.1%, *p* = 0.056) (Table 2). The majority, i.e. 75.3%, of the women with a psychiatric diagnosis after childbirth, had not been diagnosed during the five years before childbirth. In the group of CSMR women 60% (*n* = 68) with a psychiatric diagnosis had not been diagnosed before childbirth compared to 76% (*n* = 2647) of the women in the reference group (*p* < 0.001, data not shown).

**Table 2:** Distribution of psychiatric in- and outpatient care by mode of delivery in women diagnosed with one or more psychiatric disorder after delivery

Mode of delivery	Outpatient care only n (%)	Inpatient care with/without outpatient care n (%)	*P*
Caesarean section on maternal request	51 (45.1)	62 (54.9)	0.056
Other mode of delivery	1895 (54.2)	1599 (45.8)	
Total	1946 (54.0)	1661 (46.0)	

The prevalence of psychiatric disorders had increased after childbirth (3.6% vs. 5.6%, mean difference 0.2 ± 0.25, 95% CI 0.018–0.022, *p* < 0.001). There was an increase in the prevalence within both the reference group (3.5% vs. 5.5%, mean difference 0.02 ± 0.25, 95% CI 0.018–0.022, *t* = 20.250, p < 0.001) and in CSMR women (10.0% vs. 11.2%) but the increase was not significant in CSMR women (mean difference 0.01 ± 0.35, 95% CI -0.010: 0.034, *t* = 1.078, *p* = 0.281). There were significant increases in all categories of psychiatric disorders except “Organic, including symptomatic, mental disorders”, “Unspecified mental disorder” and “Disorders of psychological development” in the reference group. However the differences were small (<1%) except for “mood disorders” (mean difference 0.015 ± 0.17, 95% CI 0.013–0.016, *t* = 21.719, *p* < 0.001) and “neurotic, stress-related and somatoform disorders” (mean difference 0.016 ± 0.19, 95% CI 0.014–0.017, *t* = 20.347, *p* < 0.001). The prevalence of mood disorders had increased significantly in the group of CSMR women (mean difference 0.027 ± 0.23, 95% CI 0.012–0.041, *t* = 3.663, *p* < 0.001) but no significant differences were found for the other disorders. However new cases, i.e. women with a psychiatric diagnosis registered only after childbirth, were more common in CSMR women (6.7% vs. 4.1%, *p* < 0.001, data not shown). Being diagnosed with one or more psychiatric disorder after childbirth was more common in CSMR women (11.2% vs. 5.5%, *p* < 0.001) (Table 3). The following categories of psychiatric disorders were significantly more common in CSMR women; “Mental and behavioural disorders due to psychoactive substance use” (2.7% vs 0.8%, *p* < 0.001), “Disorders of adult personality and behavior” (1.3% vs 0.5%, p=0.001), “Behavioural syndromes associated with physiological disturbances and physical factors” (1.3% vs 0.6%, *p* = 0.003), “Neurotic, stress-related and somatoform disorders” (6.9% vs 3.1%, *p* < 0.001), “Mood disorders” (6.0% vs 2.5%, p < 0.001) and “Unspecified mental disorder” (0.3% vs 0.0%, *p* = 0.013). No significant differences were found for the other psychiatric disorders in ICD-10 (Table 3).

**Table 3:** Psychiatric disorders five years after childbirth among women in the two study groups

	Mode of delivery		
	Caesarean section on maternal request n (%)	Other delivery ^a^ n (%)	*P*
Any psychiatric disorder			
Yes	113 (11.2)	3494 (5.5)	<0.001
No	896 (88.8)	60,331 (94.5)	
Mental and behavioural disorders due to psychoactive substance use			
Yes	27 (2.7)	504 (0.8)	<0.001
No	982 (97.3)	63,321 (99.2)	
Disorders of adult personality and behaviour			
Yes	13 (1.3)	297 (0.5)	0.001**
No	996 (98.7)	63,528 (99.5)	
Behavioural syndromes associated with physiological disturbances and physical factors			
Yes	13 (1.3)	367 (0.6)	0.003
No	996 (98.7)	63,458 (99.4)	
Behavioural and emotional disorders with onset usually occurring in childhood and adolescence			
Yes	4 (0.4)	90 (0.1)	0.059 **
No	1005 (99.6)	63,735 (99.9)	
Neurotic, stress-related and somatoform disorders			
Yes	70 (6.9)	1993 (3.1)	<0.001
No	939 (93.1)	61,832 (96.9)	
Mood disorders			
Yes	61 (6.0)	1620 (2.5)	<0.001
No	948 (94.0)	62,205 (97.5)	
Mental retardation			
Yes	1 (0.1)	26 (0.0)	0.345**
No	1008 (99.9)	63,799 (100.0)	
Organic, including symptomatic, mental disorders			
Yes	2 (0.2)	29 (0.0)	0.084**
No	1007 (99.8)	63,796 (100.0)	
Schizophrenia, schizotypal, and delusional disorders			
Yes	1 (0.1)	123 (0.2)	1.0 **
No	1008 (99.9)	63,702 (99.8)	
Disorders of psychological development			
Yes	2 (0.2)	24 (0.0)	0.061**
No	1007 (99.8)	63,801 (100.0)	
Unspecified mental disorder			
Yes	3 (0.3)	29 (0.0)	0.013**
No	1006 (99.7)	63,796 (100.0)	

**Calculated by Fisher’s exact test

^a^Other delivery, including: “Vaginal delivery”, “Emergency caesarean section”, “Elective caesarean section” and “Mode of delivery missing”

CSMR increased the risk of one or more psychiatric disorder after delivery (OR 2.2, 95% CI 1.8–2.7) (Table 4). The risk was significant and increased for all categories of psychiatric disorders except “Mental retardation” (OR 2.4, 95% CI 0.3–18.0) and “Schizophrenia, schizotypal, and delusional disorders” (OR 0.5, 95% CI 0.1–3.7) (Table 4). After adjustment for potential confounders (previous psychiatric care, age, BMI, educational attainment, tobacco use, marital status, parents’ country of birth, working status and somatic diseases) the risk decreased but remained significant for “Mental and behavioural disorder due to psychoactive substance use” (aOR 2.0, 95% CI 1.3–3.1), “Neurotic, stress-related and somatoform disorders” (aOR 1.6, 95% CI 1.2–2.1), “Mood disorders” (aOR 1.7, 95% CI 1.3–2.2) and “Unspecified mental disorder” (aOR 4.0, 95% CI 1.2–13.8). The adjusted odds ratio for one or more psychiatric disorders was 1.5 (95% CI 1.2–1.9) (Table 4). There was no significant interaction effect between previous psychiatric care and CSMR. To evaluate the robustness of the estimates two different sensitivity analyses were performed. In the first analysis, all women previously diagnosed with psychiatric illness were removed from the analysis and in the second analysis no adjustments for previous psychiatric illness were included. None of these both analyses changed the parameter estimates substantially and no statistical differences in the estimates were detected. The largest risk factor for psychiatric illness five years, after childbirth was previous psychiatric care (aOR 9.9, 95% CI 8.9–10.9).

**Table 4:** Unadjusted and adjusted odds ratios for psychiatric disorders five years after childbirth by caesarean section on maternal request

Psychiatric disorder five years after childbirth	Caesarean section on maternal request			
	OR	95% CI	aOR ^a^	95% CI
Any psychiatric disorder	2.2	1.8–2.7	1.5	1.2–1.9
Mental and behavioural disorder due to psychoactive substance use	3.5	2.3–5.1	2.0	1.3–3.1
Disorders of adult personality and behaviour	2.8	1.6–4.9	1.4	0.8–2.5
Behavioural syndromes associated with physiological disturbances and physical factors	2.3	1.3–3.9	1.5	0.9–2.7
Behavioural and emotional disorders with onset usually occurring in childhood and adolescense	2.8	1.0–7.7	1.5	0.5–4.3
Neurotic, stress-related and somatoform disorders	2.3	1.8–3.0	1.6	1.2–2.1
Mood disorders	2.5	1.9–3.2	1.7	1.3–2.2
Mental retardation	2.4	0.3–18.0	0.2	0.2–3.3
Organic, including symptomatic, mental disorders	4.4	1.0–18.3	2.9	0.7–12.7
Schizophrenia, schizotypal, and delusional disorders	0.5	0.1–3.7	0.3	0.0–1.8
Disorders of psychological development	5.3	1.3–22.4	2.5	0.5–11.5
Unspecified mental disorder	6.6	2.0–21.6	4.0	1.2–13.8

^a^Adjusted for previous psychiatric disorder, age, marital status, BMI, parent’s country of birth, working status, tobacco use, educational attainment, urinary tract infection, inflammatory bowel disease, SLE, hypertension, diabetes mellitus, epilepsy, kidney disease and lung disease/asthma

## Discussion

In this study we found that CSMR is an independent risk factor for psychiatric illness after first childbirth. CSMR women more often suffer from psychiatric disorders both before and after first delivery compared to other primiparae. As in the general population, the most common disorders were “Mood disorders”, “Neurotic, stress-related and somatoform disorders” and “Mental and behavioural disorders due to psychoactive substance use” [25]. The prevalence of psychiatric disorders had increased after compared to before delivery and the majority of the women were diagnosed with a psychiatric disorder only after childbirth. When comparing within the two groups the increase in the prevalence of psychiatric disorders were significant in the reference group but not in the CSMR group except for “mood disorders”. The observed increase in the prevalence of psychiatric disorders may be caused by the fact that many of the most common psychiatric disorders increases with age, e.g. mood disorders with a median age of onset in the 4th decade [26]. The postpartum period is also a well-known risk factor for the first onset of psychiatric disorders [27]. However the increase in the prevalence of psychiatric disorders was only significant in the reference group. For most separate disorders the increase was small and not clinically significant but there was an overall increase as well as a clinically significant increase in both mood disorders and neurotic, stress-related and somatoform disorders. Since the sample size differed in the two groups, the lack of a significant increase in the CSMR group may be caused by a lack of power to detect such a difference. Furthermore the women in the reference group showed a tendency towards more often being diagnosed in the outpatient care system. Because the reporting from the outpatient care started in 2001 [18], the data before delivery (1997–2004) have a lower coverage in the outpatient care. Therefor the observed increase may merely reflect a better coverage of the outpatient care and therefore not a true increase in the prevalence. The outpatient care excludes admissions to hospital but the care is provided by specialists in psychiatry and does not include primary health care. Being diagnosed mainly in the psychiatric outpatient care may indicate that the reference group suffer from a lighter psychiatric disease burden, possible to treat in the outpatient care. These findings are in accordance with a previous study by Rouhe et al. who compared psychiatric care before and after childbirth in women with FOC or without FOC during pregnancy. They found women with FOC had an increased risk of requiring psychiatric care 5–12 years after delivery but the risk was even higher among women without FOC [14]. One speculates that the larger increase in the prevalence of psychiatric disorders after childbirth among women in the reference group may be explained by the fact that CSMR women are diagnosed and treated earlier. In Sweden women cannot choose a CS, they must meet with an obstetrician to discuss the reasons for the request and receive information on risks and benefits of CS. This meeting may lead to consultation with a psychologist/therapist. In this process the woman can be diagnosed and referred to treatment, medication and/or psychotherapy which may have improved these women’s mental health. Indeed of those women with psychiatric disorders after delivery, CSMR women were more likely to have been diagnosed before. This could indicate that CSMR women are diagnosed earlier or suffer from more complex psychiatric disorders that are relapsing in character. However the incidence of new cases with psychiatric disorders after delivery was higher among CSMR women compared to the reference group, indicating that these women still have a higher risk of psychiatric disorders also after delivery even when they were not diagnosed during pregnancy.

The prevalence of psychiatric disorders was lower than reported in previous studies [14, 25], including studies on pregnant women [15]. It is difficult to compare rates because different studies use different methods of diagnosing such as use of registers, diagnostic interviews, self-rating questionnaires and measure prevalence during different lengths of time [14, 15, 25]. The reason for the lower prevalence in our study is unknown but may be because the diagnoses in NPR are made by a physician rather than by self-rated questionnaires for example. Furthermore the coverage of NPR is incomplete [18]; and not all psychiatric disorders are diagnosed and treated in the public psychiatric healthcare system and therefore not all are registered. The coverage of the outpatient care is less valid than the inpatient care and this may have affected our results as the reference group showed a tendency towards more often be diagnosed in the outpatient care. This could make us underestimate the true prevalence of psychiatric disorders in the reference group and is a limitation in our study. However when only comparing inpatient care the prevalence of psychiatric disorders was significantly higher in CSMR women. Our inclusion criteria limited the age distribution and excluded women born outside of Sweden. These are factors associated with CSMR [3, 5, 13], and immigrants may suffer more often from psychiatric illness [28], therefore we might underestimate the frequency of CSMR and psychiatric disorders. We also lack a clear definition for CSMR. In Sweden the diagnostic code O82.8 is referred to as “CS on psychosocial indication” and is the diagnostic code that should be applied for CSMR. However some women may be misclassified as elective CS for example and thereby lower our number of cases. In our previous study we found somatic disorders such as e.g. dyspareunia increases the risk of CSMR [29]. This may be a confounding factor since these women more often report mental ill-health. However, the prevalence of dyspareunia is low and should not alter the results significantly. It is impossible to adjust for all possible somatic diseases, however this should be taken into account when interpreting the results.

Our nationwide sample with access to a range of important variables is a major strength of our study making the results more generalizable. In NPR the diagnoses are set by a physician and probably are more valid than diagnoses resulting from questionnaires that can only detect symptoms of e.g. depression or anxiety. By only including primiparae we avoided the potential confounding of a previous negative birth experience. We adjusted for sociodemographic variables, somatic diseases registered in MBR and previous psychiatric care since these variables could possibly be associated to CSMR as well as psychiatric illness. This was important since these variables were all found to have an impact on the risk of psychiatric disorders, especially previous psychiatric care (OR 13.8, 95% CI 12.6–15.2).

## Conclusion

We have previously found CSMR women more often suffer from psychiatric disorders before delivery [16] and we can now confirm that these women have an equally high prevalence of psychiatric disorders after delivery. The tendency towards a higher prevalence of inpatient care indicates these women may suffer from a heavier psychiatric disease burden. This shows that these women are a vulnerable group requiring special attention from both obstetric- and general health care providers, and this vulnerability should be taken into account when deciding on mode of delivery. The higher incidence of psychiatric disorders also indicates that these women may require a more intense follow-up. We hypothesize that these women benefit from the care during pregnancy, the incidence might have been even higher without the care. In conclusion, women giving birth by CSMR share a greater burden of mental illness before and after delivery.

## Abbreviations

BMI: Body mass index
CS: Caesarean section
CSMR women: women giving birth by caesarean section on maternal request
CSMR: Caesarean section on maternal request
FOC: Fear of childbirth
MBR: Swedish Medical Birth Registry
NPR: National patient registry
Reference group: All primiparae giving birth by other modes of delivery than caesarean section on maternal request
TPR: Total population register
